# Biological Control of Tomato Bacterial Leaf Spots and Its Impact on Some Antioxidant Enzymes, Phenolic Compounds, and Pigment Content

**DOI:** 10.3390/biology13060369

**Published:** 2024-05-23

**Authors:** Asmaa H. Akila, Mohamed A. S. Ali, Ahmed M. Khairy, Ahmed S. M. Elnahal, Haifa E. Alfassam, Hassan A. Rudayni, Fatima A. Jaber, Mohamed R. A. Tohamy

**Affiliations:** 1 Plant Pathology Department, Faculty of Agriculture, Zagazig University, Zagazig 44511, Egyptmelderiby@zu.edu.eg (M.A.S.A.);; 2 Department of Biology, College of Science, Princess Nourah bint Abdulrahman University, Riyadh 11671, Saudi Arabia; 3 Department of Biology, College of Science, Imam Mohammad Ibn Saud Islamic University, Riyadh 11623, Saudi Arabia; harudayni@imamu.edu.sa; 4 Department of Biological Sciences, College of Science, University of Jeddah, Jeddah 21589, Saudi Arabia; fajaber@uj.edu.sa

**Keywords:** biocontrol agents, culture filtrate, *Pseudomonas syringae*, tomato bacterial spot and speck, *Xanthomonas* spp.

## Abstract

**Simple Summary:**

Tomato bacterial spots and specks, caused by various strains of *Xanthomonas campestris* and *Pseudomonas syringae*, lead to significant yield losses in the El-Sharkia governorate. This study tested the efficacy of biocontrol culture filtrates from *Trichoderma* fungi (*T. viride*, *T. harzianum*, *T. album*) and bacterial agents (*Bacillus subtilis*, *Pseudomonas fluorescens*, *Serratia marcescens*) in controlling these diseases. In vitro, the culture filtrates significantly inhibited pathogenic bacterial growth. In vivo, spraying bioagents prior to infection reduced disease incidence and severity. *T. viride* was the most effective fungal bioagent, while *B. subtilis* was the top bacterial bioagent. Tomato plants that were treated with bioagents also had more phenols and chlorophyll than control plants. They also had more enzyme activity, including chitinase, peroxidase, and polyphenol oxidase. These findings suggest that biocontrol strategies could be a sustainable solution for managing tomato diseases and improving plant defense.

**Abstract:**

Tomato bacterial spots, caused by *Xanthomonas campestris* pv. *vesicatoria* (*Xcv1*) and *X. euvesicatoria* (*Xe2*), as well as bacterial specks, caused by two strains of *Pseudomonas syringae* pv. *tomato* (*Pst1* and *Pst2*), represent significant threats to tomato production in the El-Sharkia governorate, often resulting in substantial yield losses. The objective of this study was to evaluate the efficacy of various biocontrol culture filtrates, including bacteria and fungi agents, in managing the occurrence and severity of these diseases, while also monitoring physiological changes in tomato leaves, including antioxidant enzymes, phenolics, and pigment content. The culture filtrates from examined *Trichoderma* species (*T. viride, T. harzianum*, and *T. album*), as well as the tested bacteria (*Bacillus subtilis*, *Pseudomonas fluorescens*, and *Serratia marcescens*) at concentrations of 25%, 50%, and 100%, significantly inhibited the proliferation of pathogenic bacteria In vitro. For the In vivo experiments, we used specific doses of 5 mL of spore suspension per plant for the fungal bioagents at a concentration of 2.5 × 10^7^ spores/mL. The bacterial bioagents were applied as a 10 mL suspension per plant at a concentration of 1 × 10^8^ CFU/mL. Spraying the culture filtrates of the tested bioagents two days before infection In vivo significantly reduced disease incidence and severity. *Trichoderma viride* exhibited the highest efficacy among the fungal bioagents, followed by *T. harzianum* and *T. album*. Meanwhile, the culture filtrate of *B. subtilis* emerged as the most potent among the bacterial bioagents, followed by *P. fluorescens*. Furthermore, applying these culture filtrates resulted in elevated levels of chitinase, peroxidase, and polyphenol oxidase activity. This effect extended to increased phenol contents, as well as chlorophyll a, chlorophyll b, and carotenoids in sprayed tomato plants compared to the control treatment. Overall, these findings underscore the potential of these biocontrol strategies to effectively mitigate disease incidence and severity while enhancing plant defense mechanisms and physiological parameters, thus offering promising avenues for sustainable disease management in tomato production.

## 1. Introduction

Tomato (*Solanum lycopersicum* L.) holds significant agricultural value in Egypt, Africa, and globally. Egypt ranks fifth globally in potato production and boasts the largest tomato production output in Africa [1]. Moreover, tomatoes serve as a model crop for studying the growth of newly fruited plants and plant–pathogen interactions [2]. Annually, tomato cultivation faces numerous challenges, including pest infestations (such as insects and nematodes) and diseases caused by bacterial, fungal, and viral pathogens [3]. Research indicates that crop loss can vary widely depending on factors such as region, crop variety, and specific pathogens involved [4]. Hot and humid environmental conditions can exacerbate the severity of these diseases, leading to significant crop losses if pathogens are not effectively managed [5].

Bacterial diseases contribute significantly to the disease burden affecting tomato yields across various production areas in Egypt [6], including El-Sharkia governorate. These include bacterial spots, attributed to several species within the genus *Xanthomonas*, and bacterial specks, caused by *P. syringae* pv. *tomato* [7]. *Clavibacter michiganensis* induces canker, while *Ralstonia solanacearum* triggers bacterial wilt, two of the most severe bacterial diseases impacting tomato cultivation [8]. The bacterium *P. syringae* pv. *tomato* is responsible for bacterial speck, a disease highly regarded for its economic impact on tomato crops. The strain *P. syringae* pv. *tomato* strain DC 3000 serves as a model organism in molecular investigations of host–pathogen interactions [9].

Various control strategies are implemented to manage speck disease, including the utilization of biocontrol agents [9]. Pesticides commonly used to control tomato bacterial leaf spots include copper-based compounds, antibiotics, and synthetic chemicals [10]. However, they have drawbacks such as environmental pollution, harm to non-target organisms, and pathogen resistance [11]. Herbert et al. [12] reported that strains of *Xanthomonas* spp. that are resistant to streptomycin appeared after long-term use, while Gikas et al. [13] found that copper-based fungicides hurt soil microbe communities and might end up in food chains.

Biological control agents have emerged as an effective method for managing plant pathogens [14]. Researchers have documented that certain isolates of *Trichoderma* species significantly reduce plant diseases [15]. However, limited research has been conducted to assess the biocontrol efficacy of their culture filtrates against bacterial spots and speck on tomatoes. The biological control strategies of *Trichoderma* spp. involved mycoparasitism, antibiosis, competing for nutrient resources and potential infection areas, and stimulating systemic resistance within plants, as studied by [16]. Nonetheless, specific *Trichoderma* strains are recognized to excel in particular antagonistic mechanisms [17]. Hence, selecting effective *Trichoderma* strains and species is essential for specific plant pathogen control. Researchers have explored antibiotics like kasugamycin and streptomycin as controls for bacterial leaf spot and speck, particularly in greenhouse settings. Also, *Xanthomonas* spp. showed resistance to streptomycin before widespread field use [18]. Furthermore, Kasugamycin has demonstrated similar efficacy to conventional copper-mancozeb treatments against bacterial spots [19].

Recently, there has been increasing interest in alternative approaches to disease management, such as the use of biocontrol agents (BCAs). These BCAs are microorganisms or their metabolites that suppress plant pathogens through various mechanisms, including parasitism, competition, antibiosis, and induction of host defenses [20]. Culture filtrates made from BCAs have many different secondary metabolites, enzymes, and other bioactive compounds that can stop plant pathogens from growing and spreading [21].

Extensive research has focused on finding biological control methods for bacterial spots; for example, *P. syringae* Cit7 consistently provided effective control [22]. Furthermore, several plant growth-promoting rhizobacteria (PGPR), including *Bacillus pumilis*, have proven efficient in reducing bacterial spot in several field experiments [23]. PGPR plays a dual role in boosting plant growth and fighting diseases, employing tactics like hyperparasitism, antibiotic production, and systemic resistance induction. They offer a promising alternative for managing plant diseases [24]. *Bacillus* spp. is a standout among PGPRs, demonstrating potent biocontrol abilities against various plant pathogens [25]. Foliar application of *Bacillus* spp. has shown efficacy in reducing bacterial spot on tomatoes [26].

Pathogen–host interactions alter cell metabolism by affecting enzymes such as catalase, peroxidase (PO), and polyphenol oxidase (PPO). Studies reveal a significant link between bioagent efficacy and PO/PPO levels [27]. Additionally, phenolic compounds, vital secondary metabolites in plants, play key roles in functions such as pigmentation, growth, and pathogen resistance. They accumulate under stress, acting as defense compounds [28]. Phenolic production, including lignin and melanin synthesis, involves dihydroxyphenol and chinen oligomers, toxic to pathogens, bolstering plant defense [29]. PO uses a heme group to catalyze oxidation reactions with H_2_O_2_, while PPO contains Cu ions in its active site, allowing it to oxidize phenolic compounds into toxic quinones, enhancing disease resistance [30].

Microbial filtrates from soil fungi and rhizobacteria are being used more and more as biostimulants in sustainable agriculture because they can help plants grow and be more resistant to damage while lowering the need for chemicals [31]. These filtrates contain beneficial compounds that can enhance nutrient uptake, stress tolerance, and disease resistance [31]. In particular, the production of siderophores by strains like *P. fluorescens* and *B. subtilis* is notable for its iron-chelating properties, which support plant growth in nutrient-limited conditions [32].

Our study aims to evaluate the efficacy of various bacterial and fungal bioagent culture filtrates in controlling bacterial spot and speck diseases on tomato plants, both In vitro and In vivo. We hypothesize that these bioagents will inhibit the growth of pathogenic bacteria (*X. campestris* and *P. syringae* strains), leading to reduced disease incidence and severity in tomato plants. The application of these bioagents is also expected not only to reduce the incidence and severity of these diseases but also enhance the physiological defenses of tomato plants. This includes an increase in the activity of key enzymes such as chitinase, peroxidase, and polyphenol oxidase, as well as boosting levels of chlorophyll, carotenoids, and phenolic compounds. This study offers novel insights by comprehensively evaluating the efficacy of biocontrol agents against bacterial leaf spot and speck diseases on tomato plants, shedding light on their impact on key physiological parameters for sustainable disease management. Also, this approach not only controls pathogens directly but also considers the broader implications of biocontrol treatments on plant health and disease resistance. This could lead to the use of similar strategies in other crops that are affected by bacterial diseases.

## 2. Materials and Methods

The tomato bacterial leaf spot (BLS) is caused by pathogens such as *X. campestris* pv. *vesicatoria* (*Xcv1*) and *X. euvesicatoria* (*Xe2*), as well as the pathogen responsible for bacterial speck (BS), which includes two strains of *P. syringae* pv. *tomato* known as *Pst1* and *Pst2*. These pathogens were previously isolated and identified at the Plant Pathology Department, Faculty of Agriculture, Zagazig University. Their accession numbers are as follows: OR146491, OR146486, CP047072.1, and CP047071.1 for *Xcv1*, *Xe2*, *Pst1*, and *Pst2*, respectively. They were utilized in subsequent experiments.

### 2.1. Inoculum of the Pathogenic Bacteria

The previously mentioned bacterial isolates (*Xcv1*, *Xe2*, *Pst1*, and *Pst2*) were cultured in a nutrient broth medium (beef extract, yeast extract, peptone, and dextrose) on a rotary shaker for two days at 28 ± 2 °C [33]. The resulting bacterial suspension was adjusted to be 1 × 10^8^ CFU/mL using spectrophotometer OD_600_ (Spectroquant, Darmstadt, Germany) [34]. The bacterial culture filtrates were applied at a dosage of 10 mL per plant during the experiments, ensuring consistent application across all samples [33,35].

### 2.2. Bioagent Culture Filtrates

Identified fungal bioagents (*Trichoderma viride*, *T. harzianum*, and *T. album*), as well as bacterial bioagents (*Bacillus subtilis, Pseudomonas fluorescence* and *Serratia marcescens*), were obtained from Plant Pathology Dept., Fac. Agric., Zagazig Univ. Fungal bioagents were grown in 500 mL flasks containing 200 mL of Gliotoxin Fermentation Medium broth described by [36] on a rotary shaker (SK-330-Pro Scilogex) at 150 rpm for 7 days in dark room at 30 °C [37]. The broth growth was centrifuged at 10,000 rpm for 20 min, followed by filtration through Whatman No.1 filter paper and sterilization using a 0.34 μm Millipore filter. This filtered supernatant was then utilized In vitro against the tested pathogens. Likewise, metabolic substances from the bacterial isolates were obtained by cultivating the isolates in a nutrient broth medium on a rotary shaker for two days at 28 ± 2 °C. The isolates were then extracted and sterilized following the methods described by Breitling et al. [38] and Ghorbanpour et al. [39]. The effective doses used in the experiments for the fungal bioagents (*Trichoderma viride, T. harzianum*, and *T. album*) were 5 mL of spore suspension per plant at a concentration of 2.5 × 10^7^ spores/mL [40]. For the bacterial bioagents (*Bacillus subtilis*, *Pseudomonas fluorescens*, and *Serratia marcescens*), the effective dose was 10 mL of bacterial suspension per plant at a concentration of 1 × 10^8^ CFU/mL [33,35]. These doses were determined based on their efficacy in controlling tomato leaf-spot diseases and were applied consistently throughout the study to ensure reliable results.

### 2.3. In Vitro Assays

The antagonistic activity of previously purified sterile metabolites from three tested *Trichoderma* spp. and three isolates of bacterial bioagents was evaluated using three concentrations (25%, 50%, and 100%) of each metabolite. These concentrations were selected to cover a range of doses to assess the effectiveness of the metabolites against the target pathogens while ensuring that potential adverse effects or limitations associated with higher concentrations were considered. One milliliter of sterile culture filtrate from the tested bioagents was dispensed into sterilized Petri plates, followed by the addition of 1 mL containing (1 × 10^8^ CFU/mL) of each pathogen (*Xcv1*, *Xe2*, *Pst1*, *Pst2*) simultaneously. Subsequently, 15 mL of nutrient agar medium were poured into the plates. In the control treatment, sterile water was substituted for sterile metabolites. There were three replications of each treatment. The Petri plates were then incubated at 28 ± 2 °C, and data were recorded after 48 h using the cell count technique, which provides reliable counts only for live cells [41,42,43,44]. The obtained results are expressed in terms of growth percentage reduction. Results were expressed as the inhibition percentage of disease incidence and severity compared to the infected control using a formula suggested by Suresh [45], as follows:

$$Percentage\;of\;inhibition\;\left(PI\%\right)=\frac{C-T}{C}\times 100,$$

where C represents disease incidence or severity on the positive control, while T represents disease incidence or severity on the leaves treated with antagonists.

### 2.4. In Vivo Assays

We evaluated the effect of spraying tomato transplants (cv. 102) with a 100% concentration of crude extracts from three antagonistic fungi and two antagonistic bacteria. This treatment was applied two days before exposing the plants to bacterial pathogens (*Xcv1*, *Xe2*, *Pst1*, *Pst2*) with the aim of assessing its impact on disease incidence and severity. The study was conducted in a greenhouse environment with controlled conditions: 27  ±  3 °C, 68–75% relative humidity, 11 h of natural light, and 13 h of darkness daily. The plants were watered as needed to maintain soil moisture without causing waterlogging. Tomato seedlings were transplanted into 30 cm diameter plastic pots filled with 5 kg of autoclaved sandy-clay soil (1:1 sandy to clay ratio), one seedling per pot, under greenhouse conditions.

Tomato seedlings were individually sprayed two days before infection with pathogenic bacteria with sterile culture filtrates of tested bioagents [46,47]. After spraying, the seedlings were infected with bacteria and covered with polyethylene bags for 24 h to ensure infection. Following this, the bags were removed, and the plants were exposed to greenhouse conditions. For the positive control, seedlings were only sprayed with the pathogen suspension. Healthy plants were sprayed with Streptrol fungicide 21.3% as the negative control. Each treatment was replicated three times. Disease reaction was assessed two weeks later based on incidence and severity on each leaf of the infected seedlings. The obtained results are expressed in terms of inhibition % of disease incidence and severity using a formula as previously mentioned by Suresh [45].

### 2.5. Antioxidant Enzymes Activity

Enzyme extraction followed the procedure outlined by Vitória et al. [48]. To extract antioxidant enzymes, we collected 1 g of fresh tomato leaf tissue from healthy, infected, and bioagent-treated plants 10 days after treatment. Samples were kept in an icebox and transferred to the laboratory to preserve enzyme activity. Leaves were washed with distilled water to remove contaminants and surface moisture. The tissue was homogenized using a pre-chilled mortar and pestle in 2 mL of ice-cold phosphate buffer (0.1 M, pH 7.5) containing 0.5 mM EDTA to prevent metal ion interference during enzyme extraction. The homogenate was centrifuged at 15,000 rpm at 4 °C for 15 min in a Beckman refrigerated centrifuge (model Sigma 1–14, Branson-sonifier, Frankfurt, Germany), ensuring minimal heat generation. The supernatant, containing the extracted enzymes, was carefully collected and stored at −18 °C until further analysis.

#### 2.5.1. Phosphate Buffer Preparation and Enzyme Extraction

The phosphate buffer is prepared by dissolving 8.9 g of Na_2_HPO_4_ in 250 mL of distilled water in one flask (Solution A) and 3.9 g of NaH_2_PO_4_ in 250 mL of distilled water in another flask (Solution B). Solution A is then added to Solution B gradually until the pH reaches 7.1. To extract enzymes, 1 g of tomato leaves is finely crushed in 2 mL of phosphate buffer. The resulting homogenate was filtered through Whatman No.1 filter paper to remove solid debris. This process ensures that only the soluble enzyme fraction is retained for further analysis. Following filtration, the suspension is centrifuged at 6000 rpm at 4 °C for 20 min. The resulting supernatant, which contained the desired enzymes, was carefully separated and stored at −18 °C until further analysis.

#### 2.5.2. Peroxidase Activity

To assess peroxidase activity, we mixed 0.1 mL of enzyme extract with 0.5 mL of phosphate buffer (0.1 M, pH 7.1), 0.1 mL of 1% hydrogen peroxide (H_2_O_2_), and 0.3 mL of 0.05% pyrogallol solution (0.05 μL). The final volume was adjusted to 3 mL with distilled water. The reaction was initiated by adding H_2_O_2_ and monitored using a spectrophotometer (Miltonroy Spectronic 601, Ponca City, OK, USA) at a wavelength of 425 nm [49]. Absorbance readings were taken every 30 s for a total of 10 readings. The data were used to calculate peroxidase activity in milligrams per gram of fresh weight.

#### 2.5.3. Polyphenol Oxidase Activity

Enzyme samples were extracted using the described method for peroxidase activity. A tenth of the sample was mixed with 0.5 mL of sodium phosphate buffer (0.1 M, pH 7) and 0.1 mL of 0.001 N catechol. The volume was adjusted to 3 mL with distilled water. Spectrophotometer (Miltonroy Spectronic 601, USA) set at 495 nm was used to detect color density, with readings taken every 30 s for 10 intervals [50].

#### 2.5.4. Chitinase Activity

Chitinase activity was assessed using colorimetric assay [51,52]. The mixture included washed chitin, enzyme solution, and sodium acetate buffer. This mixture was incubated at 37 °C for a specific period (typically 1 h), allowing chitinase to act on the substrate. Afterward, the suspension was centrifuged to separate the enzyme products. The supernatant was treated with glucuronidase to hydrolyze chitin oligomers into Glc-NAc. Quantification of Glc-NAc was conducted following the method described by Reissig et al. [53]. Then, 0.6 M potassium tetraborate was added, followed by heating and addition of a reagent. Chitinase activity was determined using a calibration curve outlined by Boller and Metraux [54], expressing results in katals, where one katal (kat) catalyzes the formation of 1 mol of Glc-NAc per second.

### 2.6. Determination of Phenolic Compounds

Free, bound, and total phenols were quantified in tomato leaf samples from healthy, infected, and bioagent-treated plants using the colorimetric method as described by Snell and Snell [55]. For extraction, five grams of leaf tissue from each sample were collected and extracted 10 days after bioagent application. The tissues were finely chopped and submerged in 25 mL of 95% ethanol in dark brown bottles. The samples were stored at room temperature in the dark for one month to allow for complete extraction of phenolic compounds into the ethanol. Subsequently, the ethanol was evaporated using a gentle air current at room temperature until the solution was completely dry. The dried extracts were then dissolved in 10 mL of 10% isopropanol and stored in vials at 1 °C to prevent degradation of the phenolic compounds.

Total phenols were assessed by mixing 1 mL of the isopropanol extract with 0.25 mL of HCl for 10 min to release bound phenols. After cooling, 1 mL of Folin–Denis reagent and 6 mL of Na_2_CO_3_ were added, bringing the total volume to 10 mL with distilled water. The color density was measured at 520 nm using a spectrophotometer (Miltonroy Spectronic 601, USA). Tannic acid was used as a standard to generate a calibration curve, with concentrations ranging from 0 to 400 ppm, with tannic acid standards ranging from 0 to 400 ppm.

For free phenols, 1 mL of the isopropanol extract was mixed with distilled water, 1 mL of Folin–Denis reagent, and 6 mL of 20% Na_2_CO_3_ solution then adjusted to 10 mL with distilled water. The color density was measured at 520 nm following the total phenols assay. The difference between total and free phenol levels indicated bound phenol concentration.

To create a standard curve, we dissolved 1 g of catechol in distilled water to a total volume of 1 L. Using this stock solution, we prepared various concentrations in 100 mL of distilled water. Each concentration was treated with 1 mL of Folin–Denis reagent and 6 mL of Na_2_CO_3_ and diluted to 10 mL with distilled water. The optical density was measured at 520 nm, allowing us to plot a standard curve for phenol quantification.

### 2.7. Photosynthetic Pigment Analysis

For photosynthetic pigment analysis, we followed the method by Fadeel [56]. Leaf samples from healthy, infected, and bioagent-treated plants were collected, and pigments were extracted using pure acetone. To prevent loss of pigments due to oxidation, extraction was carried out quickly and samples were kept in the dark as much as possible. The pigment solution was filtered to remove solid debris.

Photosynthetic pigments, including chlorophyll a (*Chl.a*), chlorophyll b (*Chl.b*), and carotenoids (*Carto.*), were extracted from fresh leaf samples of healthy, infected, and bioagent-treated plants using pure acetone, following the method by Fadeel [56]. To prevent loss of pigments due to oxidation, the extraction was carried out quickly and samples were kept in the dark as much as possible. After extraction, the pigment solution was filtered to remove solid debris. Optical densities (OD) were measured using a spectrophotometer at wavelengths of 662 nm for (*Chl.a*), 644 nm for (*Chl.b*), and 440.5 nm for (*Carto.*). Pigment concentrations, expressed in mg/g fresh weight, were calculated using a formula adapted by [57], as follows:

*Chl.a* = (9.784 × *E*_662_) − (0.99 × *E*_644_),

*Chl.b* = (21.426 × *E*_644_) − (4.65 × *E*_662_),

*Carot.* = (4.695 × *E*_440.5_) − (0.268 × *Chl.a* − *Chl.b*),

where *E* represents optical density at the respective wavelengths. Pigment concentrations were first expressed in mg/L and then converted to mg/g fresh weight of leaves.

### 2.8. Statistical Analysis

Analysis of data was carried out using Statistix 9 software to determine the significance of various treatments via analysis of variance (ANOVA). To discern any notable differences between treatments, the LSD (least significant difference) test was utilized, with a significance threshold set at (*p* < 0.05).

## 3. Results

### 3.1. In Vitro Efficacy of Bioagents’ Filtrates on Bacterial Pathogens’ Growth Reduction

**Figure 1:**
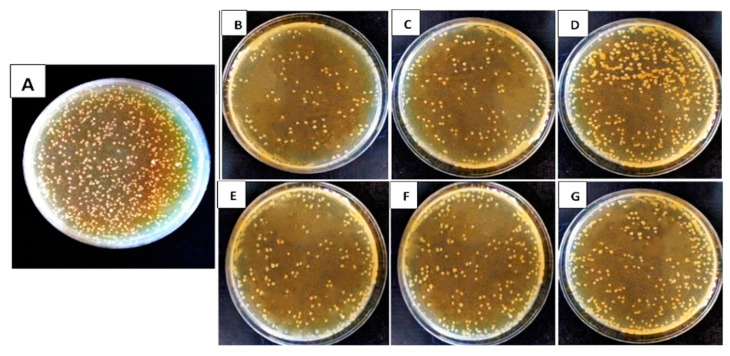
In vitro efficacy of different tested culture filtrates of tested bioagents at 100% concentration against *Xanthomonas* spp. isolate *X. campestris* pv. *vesicatoria* (*Xcv1*) growth: (**A**) Control; (**B**) *B. subtilis*; (**C**) *P. fluorescens*; (**D**) *Serratia marcescens*; (**E**) *T. viride*; (**F**) *T. harzianum*; (**G**) *T. album*.

**Figure 2:**
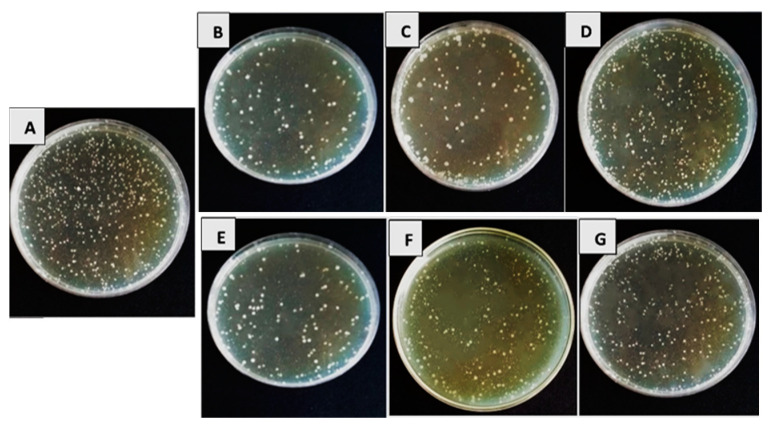
In vitro efficacy of different tested culture filtrates of tested bioagents at 100% concentration against *Pseudomonas* spp. isolate *P. syringae* pv. *tomato* (*Pst1*) growth: (**A**) control; (**B**) *B. subtilis*; (**C**) *P. fluorescens*; (**D**) *Serratia marcescens*; (**E**) *T. viride*; (**F**) *T. harzianum*; (**G**) *T. album*.

The data presented in Table 1 and Table 2, as well as Figure 1 and Figure 2, illustrate the impact of antagonistic products derived from the secondary metabolism of tested bacterial and fungal bioagents on the percentage reduction in growth of the targeted bacterial pathogens. The results clearly showed that the tested bioagents significantly slowed down the growth of pathogenic strains of *Xanthomonas* spp. and *Pseudomonas* spp. compared to the control treatment. The recorded data represent the percentage reduction in growth achieved.

At the highest concentration of 100%, the filtrates of *B. subtilis* demonstrated remarkable inhibition, resulting in the highest percentage reduction in bacterial pathogen growth (71.8%, 63.4%, 71.1%, and 66.7% for the tested pathogens *Xcv1*, *Xe2*, *Pst1*, and *Pst2*, respectively). Following closely, *P. fluorescens* exhibited notable inhibition as well, with reductions of 69.1%, 63.4%, 68.8%, and 65.1% against the respective pathogens. Conversely, *S. marcescens*, even at higher concentrations, showed comparatively lower effectiveness, achieving reductions of 43.8%, 36.7%, 47.1%, and 43.3% against the tested pathogens.

Table 2 also shows the outcomes of antagonistic culture filtrates made from secondary metabolic products from *T. viride*, *T. harzianum*, and *T. album*. We observed significant growth inhibition of *Xanthomonas* spp. and *Pseudomonas* sp. strains compared to the control treatment, where no reduction was evident. *Trichoderma viride* demonstrated the highest percentage growth reduction against all tested pathogens at the highest concentration (100%), with values of 73.7%, 71.0%, 68.0%, and 59.6%, respectively. The effectiveness followed a descending order of *T. viride*, *T. harzianum*, and *T. album*. We noted the differences in concentrations and selected 100% to evaluate their impact on tomato seedlings under greenhouse conditions. Conversely, the limited effectiveness of *S. marcescens* in inhibiting the growth of pathogenic bacteria suggests that it is unsuitable for use in greenhouse conditions. Figure 1 shows the In vitro effectiveness of various culture filtrates of bioagents against *Xcv1* isolate growth. Similarly, Figure 2 illustrates the In vitro efficacy of the same culture filtrates against *Pst1* isolate growth.

### 3.2. In Vivo Assessment

#### 3.2.1. Efficacy of Bioagents’ Culture Filtrates on Leaf Spot and Speck Diseases in Tomato Plants

**Figure 3:**
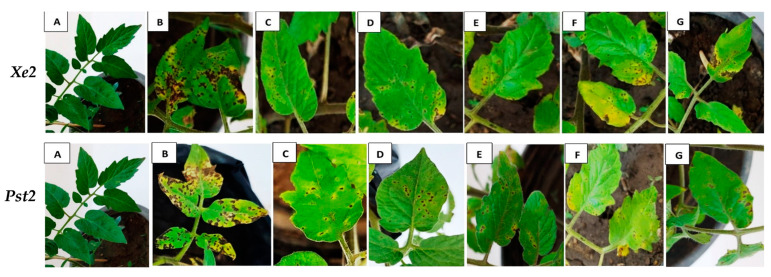
In vivo efficacy of different tested secondary metabolites (culture filtrate) of bacteria at 100% against *X. euvesicatoria* (*Xe2*) and *P. syringe* pv. *tomato* (*Pst2*). Plants were treated with biological products two days before infection. The negative control was treated with Streptrol fungicide 21.3% at the same time, while the healthy control received no treatment and was not infected. (**A**) Healthy control; (**B**) infected control; (**C**) infected treated with *B. subtilis*; (**D**) infected treated with *P. fluorescens*; (**E**) infected treated with *T. viride*; (**F**) infected treated with *T. harzianum*; (**G**) infected treated with *T. album*.

The In vivo efficacy of tested secondary metabolites (100% culture filtrates) from bacterial and fungal bioagents on disease incidence and severity is analyzed in Table 3 and Figure 3. In most cases studied, *B. subtilis* emerged as the most effective inhibiting bioagent, resulting in a significant percentage decrease in disease incidence compared to *P. fluorescens*. Specifically, *B. subtilis* achieved reductions of 56.9%, 54.8%, 41.7%, and 34.9% in disease incidence caused by *Xcv1*, *Xe2*, *Pst1*, and *Pst2* bacterial pathogens, respectively, compared to the positive infected control. Meanwhile, *P. fluorescens* achieved reductions of 43.0%, 39.7%, 34.9%, and 33.4% against the respective pathogens.

Similarly, in terms of disease severity, *B. subtilis* demonstrated significant reductions of 65.4%, 60.0%, 56.6%, and 51.5% for *Xcv1*, *Xe2*, *Pst1*, and *Pst2*, respectively. Interestingly, disease severity was more affected by the tested bacterial bioagents compared to disease incidence. Among the *Trichoderma* spp. bioagents, *T. viride* was the most effective, achieving reductions of 53.8%, 50.8%, 47.7%, and 39.5% in disease incidence for *Xcv1*, *Xe2*, *Pst1*, and *Pst2*, respectively. Its effect on disease severity was also notable, recording reductions of 58.3%, 52.3%, 51.1%, and 41.5% for *Xcv1*, *Xe2*, *Pst1*, and *Pst2*, respectively, compared to the positive control. The effectiveness of *T. viride* was followed by *T. harzianum* and *T. album* in descending order.

#### 3.2.2. In Vivo Changes in Phenolic Compounds

The results obtained in Table 4 indicate that *P. fluorescens* had a more significant impact on increasing phenol contents in the leaves, including total phenols, free phenols, and bound phenols, compared to *B. subtilis*. Similarly, among the *Trichoderma* spp., *T. viride* exhibited the highest efficacy in enhancing phenol contents (total, free, and bound), followed by *T. harzianum* and *T. album*, in comparison with the untreated control. It is noteworthy that the phenol contents in infected tomato seedlings treated with the tested bioagents exhibited higher enzymatic activity values compared to untreated infected tomato seedlings. This suggests that the application of bioagents resulted in increased activity of enzymes associated with phenol metabolism in response to infection.

#### 3.2.3. In Vivo Changes in Antioxidant Enzymes

The results presented in Table 5 show that *B. subtilis* exhibited the highest increase in the enzyme activities of chitinase, peroxidase, and polyphenol oxidase in tomato plants infected by the bacterial pathogens *Xcv1*, *Xe2*, *Pst1*, and *Pst2*, compared to *P. fluorescens*. In turn, *T. viride* was had most effective fungal bioagent on activity of chitinase, peroxidase, and polyphenol oxidase, followed by *T. harizianum* and *T. album*, compared with the untreated infected control. Notable variations in enzyme activity were observed across treatments. The most effective bioagents, ranked based on their impact on enzyme activity, were *B. subtilis*, *T. viride*, *P. fluorescens*, *T. harzianum*, and *T. album*. These bioagents exhibited significant differences (*p* < 0.05) in enzyme activity compared to the infected control group.

#### 3.2.4. Changes in Chlorophyll a, Chlorophyll b and Carotenoids

The data obtained in Table 6 revealed that *B. subtilis* exhibited the most pronounced inhibition against tomato bacterial spot and speck diseases, leading to a significant increase in *Chl.a*, *Chl.b*, and *Carto*., followed by *P. fluorescens*. Similarly, among the *Trichoderma* spp., *T. viride* demonstrated the most significant effectiveness in enhancing *Chl.a*, *Chl.b*, and *Carto*., followed by *T. harzianum* and *T. album* at a concentration of 100%, when compared with the untreated control.

## 4. Discussion

Managing bacterial spots or specks on tomatoes poses challenges due to the limited availability of effective chemical control measures and commercially resistant varieties. Breeding tomatoes for resistance to bacterial spots and specks faces complexity due to various factors, including the emergence of new pathogen species capable of overcoming existing resistance traits [58]. In recent decades, various strategies have been proposed to encourage the application of copper-based agrochemicals in the treatment of diseases caused by various *Xanthomonas* species. These agrochemicals partially or completely mitigate the disease caused by *P. syringe* pv. *tomato*, reducing the harmful effects of chemical use on consumers and the environment. Among these alternatives, biocontrol stands out as an effective, environmentally friendly, and economical solution [59].

The In vitro study showed that the culture filtrate supernatant (CFS) from the secondary metabolites of *B. subtilis* significantly inhibited the growth of pathogenic *Xanthomonas* spp. (*Xcv1* and *Xe2*) and *Pseudomonas* spp. strains (*Pst1* and *Pst2*) in Petri plates. The highest inhibition of growth was observed at the maximum CFS concentrations from *B. subtilis* and *P. fluorescens*. In contrast, *S. marcescens* was the least effective bioagent against the pathogenic bacteria, so it was not included in further tests on tomato seedlings under In vivo conditions. Among the *Trichoderma* species, *T. viride* showed the greatest inhibition effect, followed by *T. harzianum* and *T. album*. Studies suggest that these variations in the In vitro efficacy might result from differences in the composition and concentration of cell-free supernatants, including antibiotics [60], lytic enzymes, and siderophores [61].

Culture filtrates (CF) from biocontrol agents have also shown effectiveness in reducing plant pathogens in a range of other crops. For example, culture filtrates from *Bacillus amyloliquefaciens* RS-25, *B. subtilis* Z-14, *B. licheniformis* MG-4, and *B. subtilis* Pnf-4 have been found to suppress gray mold caused by *Botrytis cinerea* on postharvest fruits, including tomato, grapefruit, and strawberry [62]. Culture filtrates of *Trichoderma* species, including *T. harzianum, T. longibrachiatum,* and *T. atroviride*, were shown to inhibit *Alternaria solani* in tomato plants (variety: doucen), demonstrating potential for reducing early blight symptoms under greenhouse and field conditions. The CF-treated plants showed higher antioxidant enzymes, higher flavonoids, and increased phenolic compounds [63]. Moreover, *Trichoderma virens* and *Bacillus velezensis* culture filtrates have exhibited antimicrobial activity against *Ralstonia solanacearum* in tomato plants, reducing the severity of bacterial wilt [64].

Upon reaching the surface of tomato leaves, bacterial cells commence colonization of the phyllosphere, a process previously observed via confocal laser detection and scanning microscopy [65]. These bacteria can inhabit various epidermal cell structures, like stomata, trichomes, lenticels, and hydathodes, through which they penetrate the host plant. Pathogens responsible for bacterial spots or specks typically enter the leaf apoplast via stomata, multiplying in the intercellular and substomatal spaces. As the disease progresses, invasion by bacterial pathogens and the subsequent degradation of chlorophylls (*Chl.a*, *Chl.b*, and *Carto*.) within plant cells lead to widespread necrosis, resulting in the loss of phyllosphere individuals. Application of culture filtrates (CFSs) as a bioactive agent has been shown to mitigate these symptoms in infected plants.

The In vivo current data revealed that sprayed *B. subtilis* CFSs significantly decreased the percentage of disease incidence and severity, followed by *P. fluorescence*, compared with control treatment. Among the *Trichoderma* spp., *T. viride* exhibited the highest efficacy in reducing both disease incidence and severity in sprayed infected tomato plants, followed by *T. harzianum* and *T. album*. The success of our study in preventing tomato spot infections may be attributed to various scientific theories elucidated by numerous investigations. According to [66], there has been a notable rise in the utilization and prevalence of *Bacillus* spp. with antagonistic properties for disease management. *Bacillus* spp. possess distinctive attributes, including rapid replication rates, resilience to adverse environmental conditions, and a wide-ranging capacity for biocontrol. Certain *Bacillus* species possess an exceptional capacity to withstand unfavorable environmental circumstances and reproduce quickly, giving them a wide range of biocontrol capabilities. *Bacillus* enhances plant resistance by producing antimicrobial antibiotics, toxins, hydrolytic enzymes, and lipopeptides [67]. Many antibiotics produced by *B. subtilis* have been proven to be broad-spectrum antibiotics, such as glycopeptides, which play a role in stimulating plant growth [68]. Specific strains of *B. subtilis* were documented to efficiently suppress *Ralstonia* wilt in particular plant hosts at specified concentrations [69]. Though *B. subtilis* is known for its production of various antimicrobial agents, including a wide array of lipopeptides, it also functions as a potent biosurfactant [70]. *Pseudomonas* spp. are strong candidates for biocontrol due to their fast growth, adaptability to various environments, and broad antibiotic synthesis capability [71]. These studies corroborate and align with the findings of our study.

Another explanation provided in this study categorizes the biocontrol bacteria utilized as members of a group known as (PGPB) or plant-promoting rhizobacteria (PGPR), primarily as a large number of them come from the rhizosphere [72]. Rhizobacterial communities often include *Pseudomonas* and *Bacillus* strains, which can encourage the development of plants and act as efficient biocontrol agents. Plants can develop systemic resistance to *Bacillus* species, which can also compete for habitats and hinder phytopathogens [73]. The primary biocontrol mechanism involves producing secondary metabolites that inhibit pathogens [74]. Commonly used biological control agents include multiple strains of *Bacillus* and *Pseudomonas* [71]. These bacteria directly combat plant pathogens by releasing bioactive compounds. Alternatively, they can act indirectly by inducing plant defense responses, as seen in the *B. subtilis* group [75]. Additionally, they form beneficial connections with plant roots, releasing biomolecules with antibiotic properties [76]. *Pseudomonas* spp., typically found in soil and belonging to the *P. putida* group [77], produce Xantholycine, a secondary metabolite with anti-iron oxidation activity that inhibits the growth of various pathogenic bacteria [77].

*Trichoderma*, a soil fungus used as a bioagent in this study, exhibits antagonistic properties against plant pathogens [78]. Its mechanisms include competition, mycoparasitism, and antibiosis [79]. Antibiosis involves releasing chemical products that inhibit pathogen growth, often through extracellular enzymes and antibiotics [80]. *Trichoderma* spp. also produces secondary metabolites like polyketides, pyrones, and terpenes with antifungal and antibacterial properties [81]. Crude extracts of *T. harzianum* have been shown to effectively inhibit various plant pathogenic bacteria at different concentrations [80].

Our findings demonstrate that the bacteria employed In vitro effectively inhibited the growth of *Xcv1* and *Xe2*, as well as *Pst1* and *Pst2*, effectively controlling tomato bacterial leaf spot and speck when their culture filtrates were applied to infected tomato seedlings. This effect may be attributed to the production of siderophores, amylases, and proteases. Siderophores and lytic enzymes contribute to competitive colonization and biological control within plant tissues. Additionally, siderophores may induce both systemic and local host resistance [82]. The ability of fluorescent dyes to create proteases and siderophores could contribute to their efficacy against bacterial pathogens responsible for spot formation [83].

The timing of biological agent application is critical for effective plant disease control. Pre-inoculation studies, like those by Ons et al. [84] and Scortichini [85], demonstrated that applying biocontrol agents a few days before pathogen exposure can reduce disease severity by promoting early colonization and resistance. Post-inoculation applications, as investigated by Wei et al. [86], showed that treating plants after infection can suppress disease progression by enhancing plant defense mechanisms. Simultaneous applications, as evidenced by Ajuna et al. [87], indicated that introducing biological agents at the same time as pathogens can reduce disease symptoms through direct competition and disruption of pathogen establishment.

Using crude secondary metabolites or culture filtrates (CFS) offers advantages over using the microorganisms that produce them, as they do not require the same level of competition and colonization in the plant’s environment. However, there are concerns regarding the limited representation of the genetic diversity available for synthesizing bioactive compounds, particularly with antibiotics from bacteria like *B. amyloliquefaciens*. While some antibiotics may reach efficient concentrations in the rhizosphere, others may not, posing challenges. Moreover, high concentrations of secondary metabolites may pose risks to crops and the environment, necessitating the identification of safe compounds or those effective at lower concentrations. Selecting the optimal application strategy is also crucial for success [88].

Microbial filtrates, especially those from strains like *P. fluorescens* and *B. subtilis*, are gaining attention for their role as biostimulants in agriculture [89]. These filtrates contain a variety of biologically active compounds, including siderophores, which are crucial for iron chelation, enhancing plant growth, and improving resistance to environmental stresses [89]. Siderophores facilitate iron uptake in plants, an essential micronutrient that plays a critical role in cellular processes and chlorophyll synthesis [90]. Recent studies have shown that the application of microbial filtrates not only boosts nutrient uptake but also activates soil and plant-associated beneficial microbiota [90]. This dual action enhances plant resilience against pathogens and abiotic stresses, promoting a more sustainable agricultural practice by reducing the reliance on chemical fertilizers and pesticides [91].

The cultural filtrates of the tested bioagents increased total phenols, chitinase, PO, and PPO activities in tomato leaves infected by *Xanthomonas* spp. and *Pseudomonas* strains compared to untreated infected plants. These findings suggest that spraying bioagent culture filtrates can enhance key defense mechanisms in tomato plants. Treated plants exhibited higher enzymatic activity, indicating an improved ability to combat plant pathogens. Oxidative enzymes like PO and PPO play a critical role in forming lignin and reinforcing cell structure, thus aiding in plant defense [92].

Phenolic compounds, which are among the most abundant secondary metabolites in plants, regulate products governing disease resistance in crops [93]. Studies show a positive correlation between higher phenolic content and increased plant disease resistance. Phenolic compounds, present ubiquitously in plants, are essential in various aspects of plant life, particularly in interactions with the environment [94]. Certain phenolics trigger plant defense responses against pathogens.

Infected pathogens significantly affect chloroplasts within plant cells, leading to visible symptoms on tomato leaves, such as bacterial spots or specks. These symptoms typically appear as dark, irregular, or circular water-soaked spots with chlorotic halos, as observed in the unsprayed control plants. Lesion growth exacerbates pigment degradation, reducing green photosynthetic tissue. Leaves from untreated infected plants showed increased yellowing and merging lesions, indicating reduced concentrations of key pigments like (*Chl.a*, *Chl.b*, *Carto.*), which are critical for light absorption and photochemical reactions [95]. The presence of chlorotic halos, a sign of chloroplast degradation, suggests that infections by *Xanthomonas* spp. and *Pseudomonas* strains might damage the thylakoid network, impairing CO_2_ fixation and electron transport [96].

Applying culture filtrates from the tested bacteria and fungi, which contain antimicrobial substances like extracellular antibiotics, polyketides, pyrones, and terpenes, to tomato seedling leaves two days before infection inhibits bacterial growth. These substances act by preventing bacterial pathogens from entering through stomatal openings, which reduces their ability to proliferate in the intercellular spaces, where they could infect leaf cells and degrade pigments such as carotenoids and chlorophyll. As a result, the treated plants showed higher concentrations of (*Chl.a*, *Chl.b*, and *Carto.*) compared to plants that were infected but not treated with culture filtrates. The improved pigment levels in treated plants suggest that the bioagents effectively prevent infection by blocking bacterial entry through the stomata.

## 5. Conclusions

This study highlights the significant threat posed by bacterial spot diseases to tomato production, emphasizing the urgent need for effective management strategies to safeguard yield and fruit quality. Among the microbial groups tested, *T. viride* stood out as the most effective fungal bioagent, primarily due to its robust antipathogenic activity and ability to significantly enhance plant defense enzymes. Its superiority could be attributed to its aggressive colonization and higher competitive saprophytic ability, which makes it an excellent candidate for biocontrol applications. For the bacterial bioagents*, B. subtilis* emerged as the most potent, possibly due to its ability to produce a wide range of antibiotics and induce systemic resistance within the plant. This bacterial species showed not only high efficacy in reducing disease severity but also enhanced the biochemical pathways involved in plant defense, as evidenced by increased levels of peroxidase and polyphenol oxidase activities. The results suggest that preemptive application of these biocontrol agents—specifically, two days prior to potential pathogen attack—provides an optimal window for the agents to establish themselves and activate plant defense mechanisms. This timing is crucial for ensuring that the plants are sufficiently primed against incoming pathogens, thereby reducing the incidence and severity of the diseases. Overall, our study not only highlights the potential of using *Trichoderma* and *Bacillus* species as biocontrol agents but also provides a foundation for further research into their practical applications in sustainable agriculture. This approach promises not only to control disease but also to enhance the overall health and productivity of tomato plants, paving the way for more resilient agricultural practices.

## Figures and Tables

**Table 1:** In vitro efficacy of different bacterial bioagent culture filtrates and their concentrations against *X. campestris* pv. *vesicatoria* (Xcv*1*), *X. euvesicatoria* (*Xe2*), and two strains of *P. syringae* pv. *tomato* (*Pst1* and *Pst2*), measured as percentage growth reduction.

Pathogen	Bioagent	% Reduction		
		25%	50%	100%
*X. campestris* pv. *vesicatoria* (*Xcv1*)	*B. subtilis*	41.1 l	56.3 gh	71.8 a
	*P. fluorescens*	27.3 pqr	54.5 h	69.1 bc
	*S. marcescens*	25.7 r	36.6 m	43.8 k
*X. euvesicatoria* (*Xe2*)	*B. subtilis*	31.6 n	55.0 h	63.4 f
	*P. fluorescens*	17.9 t	42.0 kl	63.4 f
	*S. marcescens*	20.5 s	28.6 pq	36.7 m
*P. syringae* pv. *tomato* (*Pst1*)	*B. subtilis*	27.4 pqr	64.1 f	71.1 ab
	*P. fluorescens*	29.3 op	58.3 g	68.8 cd
	*S. marcescens*	26.4 qr	31.0 no	47.1 j
*P. syringae* pv. *tomato* (*Pst2*)	*B. subtilis*	21.6 s	51.0 i	66.7 de
	*P. fluorescens*	14.2 u	63.7 f	65.1 ef
	*S. marcescens*	21.8 s	25.4 r	43.3 k
LSD	Bacteria	2.84		
	Bioagent	1.96		
	bacteria × bioagent	1.81		

The assigned letters represent Duncan’s multiple range test outcomes at *p* < 0.05 significance. Different letters signify significant treatment differences, while similar ones denote non significance. ‘×’ indicates variable interaction.

**Table 2:** In vitro efficacy of different *Trichoderma* spp. culture filtrates and their concentrations against *X. campestris* pv. *vesicatoria* (*Xcv1*), *X. euvesicatoria* (*Xe2*), and two strains of *P. syringae* pv. *tomato* (*Pst1* and *Pst2*), measured as percentage growth reduction.

Pathogen	Bioagent	%Reduction		
		25%	50%	100%
*X. campestris* pv. *vesicatoria* (*Xcv1*)	*T. viride*	43.1 i	52.9 g	73.7 a
	*T. harzianum*	25.7 m	30.4 kl	59.3 e
	*T. album*	20.3 n	29.3 l	41.9 i
*X. euvesicatoria* (*Xe2*)	*T. viride*	55.4 f	64.5 d	71.0 b
	*T. harzianum*	21.6 n	32.5 k	46.3 h
	*T. album*	16.9 o	28.6 l	36.6 j
*P. syringae* pv. *tomato* (*Pst1*)	*T. viride*	46.6 h	51.2 g	68.0 c
	*T. harzianum*	30.4 kl	46.8 h	55.9 f
	*T. album*	28.9 l	37.7 j	42.5 i
*P. syringae* pv. *tomato* (*Pst2*)	*T. viride*	28.9 l	45.6 h	59.6 e
	*T. harzianum*	17.2 o	24.6 m	43.1 i
	*T. album*	17.3 o	20.1 n	31.9 k
LSD	Bacteria	3.34		
	Bioagent	2.31		
	bacteria × bioagent	2.13		

The letters denote the results of the Duncan’s multiple range test with a significance level of *p* < 0.05. Distinct letters indicate significant variations in treatment, whereas similar letters indicate negligible relevance. ‘×’ indicates variable interaction.

**Table 3:** In vivo efficacy of different tested secondary metabolites (culture filtrates) of different bacteria and fungi bioagents at 100% concentration on disease incidence (DI) and disease severity (DS) of leaf spot and speck disease in tomato plants.

Pathogen	Bioagent	%Reduction	
		DI%	DS%
*X. campestris* pv. *vesicatoria* (*Xcv1*)	*B. subtilis*	56.9 gh	65.4 j
	*P. fluorescens*	43.0 b–f	46.5 c–g
	*T. viride*	53.8 fgh	58.3 hi
	*T. harzianum*	48.8 def	55.0 f–i
	*T. album*	43.1 b	46.8 ghi
	Infected	100.0 a	90.5 ab
	Healthy	0.0 i	0.0 k
*X. euvesicatoria* (*Xe2*)	*B. subtilis*	54.8 d–h	60.0 hij
	*P. fluorescens*	39.7 bc	43.6 cd
	*T. viride*	50.8 d–g	52.3 e–i
	*T. harzianum*	45.4 bcde	52.7 e–i
	*T. album*	36.7 bcd	41.4 d–h
	Infected	100.0 a	95.4 a
	Healthy	0.0 i	0.0 k
*P. syringae* pv. *tomato* (*Pst1*)	*B.subtilis*	41.7 fgh	56.6 ij
	*P. fluorescens*	34.9 c–f	39.5 c–f
	*T. viride*	47.7 h	51.1 ghi
	*T. harzianum*	43.5 fgh	53.3 hi
	*T. album*	40.7 e–h	44.8 g–h
	Infected	95.0 a	80.0 b
	Healthy	0.0 i	0.0 k
*P. syringae* pv. *tomato* (*Pst2*)	*B. subtilis*	34.9 bcde	51.5 d–h
	*P. fluorescens*	33.4 bcd	37.1 c
	*T. viride*	39.5 b–f	41.5 cd
	*T. harzianum*	50.0 def	40.5 d–h
	*T. album*	38.4 b–f	39.8 cd
	Infected	100.0a	85.5 ab
	Healthy	0.0 i	0.0 k
LSD	Bacteria	4.28	3.99
	Bioagent	5.67	5.27
	bacteria × bioagent	11.33	10.55

The letters refer to Duncan’s multiple range test outcomes at *p* < 0.05 significance. Dissimilar letters indicate significant differences, whereas similar ones indicate non significance. ‘×’ denotes the interaction between variables.

**Table 4:** Activity of total phenol contents measured as mg/g fresh weight in leaves of infected tomato plant cv 102 by *X. campestris* pv. *vesicatoria* (*Xcv1*), *X. euvesicatoria* (*Xe2*), and two strains of *P. syringae* pv. *tomato* (*Pst1*) and (*Pst2*) on treated plants with bacteria and fungi bioagents culture filtrates at 100% concentration.

Bioagent	Pathogen	Free Phenol	Bound Phenol	Total Phenol
Infected control	Healthy control	1.25 o	0.50 v	1.75 s
	*X.campestris* pv. *vesicatoria* (*Xcv1*)	1.55 gh	0.68 s	2.23 mn
	*X. euvesicatoria* (*Xe2*)	1.51 ij	0.65 tu	2.17 o
	*P. syringae* pv. *tomato* (*Pst1*)	1.52 hij	0.66 st	2.18 no
	*P. syringae* pv. *tomato* (*Pst2*)	1.51 ijk	0.66 tu	2.16 o
*B. subtilis*	Healthy control	1.25 no	0.64 u	1.90 r
	*X. campestris* pv. *vesicatoria* (*Xcv1*)	1.46 l	0.78 o	2.24 m
	*X. euvesicatoria* (*Xe2*)	1.48 kl	0.80 n	2.27 lm
	*P. syringae* pv. *tomato* (*Pst1*)	1.49 jkl	0.81 mn	2.30 kl
	*P. syringae* pv. *tomato* (*Pst2*)	1.50 ijk	0.82 m	2.32 jk
*P. fluorescens*	Healthy control	1.27 mn	0.70 r	1.98 q
	*X. campestris* pv. *vesicatoria* (*Xcv1*)	1.55 gh	0.88 i	2.43 gh
	*X. euvesicatoria* (*Xe2*)	1.55 fgh	0.89 hi	2.44 g
	*P. syringae* pv. *tomato* (*Pst1*)	1.57 efg	0.90 gh	2.47 fg
	*P. syringae* pv. *tomato* (*Pst2*)	1.58 efg	0.91 g	2.49 ef
*T. viride*	Healthy control	1.26 no	0.66 st	1.92 r
	*X. campestris* pv. *vesicatoria* (*Xcv1*)	1.51 ij	0.84 kl	2.35 ij
	*X. euvesicatoria* (*Xe1*)	1.50 ijk	0.84 k	2.34 ijk
	*P. syringae* pv. *tomato* (*Pst1*)	1.53 hi	0.86 j	2.38 hi
	*P. syringae* pv. *tomato* (*Pst2*)	1.53 hij	0.86 j	2.38 i
*T. harzianum*	Healthy control	1.31 m	0.73 q	2.04 cd
	*X. campestris* pv. *vesicatoria* (*Xcv1*)	1.59 de	0.93 f	2.52 de
	*X. euvesicatoria* (*Xe2*)	1.59 def	0.93 df	2.52 de
	*P. syringae* pv. *tomato* (*Pst1*)	1.60 b-e	0.95 de	2.55 cd
	*P. syringae* pv. *tomato* (*Pst2*)	1.60 cde	0.95 d	2.55 cd
*T. album*	Healthy control	1.33 m	0.76 p	2.09 s
	*X. campestris* pv. *vesicatoria* (*Xcv1*)	1.61 bcd	0.97 c	2.58 bc
	*X. euvesicatoria* (*Xe2*)	1.64 ab	0.99 b	2.62 ab
	*P. syringae* pv. *tomato* (*Pst1*)	1.63 abc	0.99 b	2.62 ab
	*P. syringae* pv. *tomato* (*Pst2*)	1.66 a	1.01 a	2.66 a
LSD	Bacteria	0.01	6.25	0.01
	Bioagent	0.01	6.51	0.02
	bacteria × bioagent	0.04	0.02	0.05

The letters indicate Duncan’s multiple range test outcomes at *p* < 0.05 significance. Similar letters represent non significance among treatments, while different letters imply significant differences. The symbol ‘×’ indicates the interaction between variables.

**Table 5:** Effect of sprayed different bioagents culture filtrates at 100% concentration on enzymes activity of infected tomato plants cultivars (102) by *Xanthomonas campestris* pv. *vesicatoria* (*Xcv1*), *Xanthomonas euvesicatoria* (*Xe2*), and two strains of *Pseudomonas syringae* pv. *tomat*o (*Pst1* and *Pst2*) measured as Mg/g leaves.

Bioagent	Pathogen	Chitinase	Polyphenol Oxidase	Peroxidase
Infected control	Healthy control	0.06 t	30.63 b	49.0 n–q
	*X. campestris* pv. *vesicatoria* (*Xcv1*)	0.07 st	32.39 c	51.8 pq
	*X. euvesicatoria* (*Xe2*)	0.08 rs	34.52 ab	55.2 l–p
	*P. syringae* pv. *tomato* (*Pst1*)	0.08 r	35.21 d	56.3 q
	*P. syringae* pv. *tomato* (*Pst2*)	0.09 q	39.54 a	63.3 l–p
*B. subtilis*	Healthy control	0.21 mn	0.25 abc	64.5 n–q
	*X. campestris* pv. *vesicatoria* (*Xcv1*)	0.31 j	0.31 e	65.8 b–f
	*X. euvesicatoria* (*Xe2*)	0.35 h	0.35 e	66.5 ab
	*P. syringae* pv. *tomato* (*Pst1*)	0.46 cd	0.46 e	71.8 g–k
	*P. syringae* pv. *tomato* (*Pst2*)	0.51 a	0.51 e	78.1 abc
*P.fluorescens*	Healthy control	0.24 o	0.24 abc	60.4 n–q
	*X. campestris* pv. *vesicatoria* (*Xcv1*)	0.28 j	0.28 e	61.8 f–j
	*X. euvesicatoria* (*Xe2*)	0.31 i	0.31 e	62.5 b–f
	*P. syringae* pv. *tomato* (*Pst1*)	0.45 e	0.45 e	67.4 k–o
	*P. syringae* pv. *tomato* (*Pst2*)	0.45 de	0.45 e	73.2 c–g
*T.viride*	Healthy control	0.28 k	0.28 abc	62.6 n–q
	*X. campestris* pv. *vesicatoria* (*Xcv1*)	0.30 h	0.30 e	63.6 d–h
	*X. euvesicatoria* (*Xe2*)	0.46 bc	0.46 e	64.6 a–d
	*P. syringae* pv. *tomato* (*Pst1*)	0.355 h	0.35 e	69.7 l–m
	*P. syringae* pv. *tomato* (*Pst2*)	0.47 b	0.47 e	75.5 a–e
*T. harzianum*	Healthy control	0.25 n	0.25 abc	58.4 n–q
	*X. campestris* pv. *vesicatoria* (*Xcv1*)	0.26 lm	0.26 e	59.3 h–l
	*X. euvesicatoria* (*Xe2*)	0.26 l	0.26 e	60.1 e–h
	*P. syringae* pv. *tomato* (*Pst1*)	0.44 f	0.44 e	64.7 m–p
	*P. syringae* pv. *tomato* (*Pst2*)	0.45 e	0.45 e	71.0 g e–i
*T. album*	Healthy control	0.21 p	0.21 abc	56.2 n–q
	*X. campestris* pv. *vesicatoria* (*Xcv1*)	0.24 o	0.24 e	57.7 j–n
	*X. euvesicatoria* (*Xe2*)	0.26 l	0.26 e	58.3 g–j
	*P. syringae* pv. *tomato* (*Pst1*)	0.41 g	0.41 e	62.8 o–q
	*P. syringae* pv. *tomato* (*Pst2*)	0.43 f	0.43 e	68.4 jg–k
LSD	bacteria	2.47	0.27	1.33
	bioagent	2.99	0.80	1.14
	bacteria × bioagent	8.27	0.90	4.47

The assigned letters (‘a’, ‘b’, ‘c’, ‘d’, …) represent Duncan’s multiple range test outcomes at *p* < 0.05 significance. Different letters imply significant differences, while similar ones denote non significance. The ‘×’ indicates the interaction between variables.

**Table 6:** Effect of different bioagents on chlorophyll a, b, and carotenoids contents in infected tomato plants by *X. campestris* pv. *vesicatoria* (*Xcv*), *X. euvesicatoria* (*Xe*), and two strains of *P. syringae* pv. *tomato* (*Pst1* and *Pst2*) measured as Mg/g leaves.

Bioagent	Pathogen	Chlorophyll a Mg/g Leaves	Chlorophyll b Mg/g Leaves	Carotenoids Mg/g Leaves
Infected control	Healthy control	1.51 j	0.80 c–g	1.07 h
	*X. campestris* pv. *vesicatoria* (*Xcv1*)	1.62 hij	0.85 c–g	1.15 fgh
	*X. euvesicatoria* (*Xe2*)	1.61 hij	0.85 d–g	1.15 fgh
	*P. syringae* pv. *tomato* (*Pst1*)	1.57 hij	0.85 fg	1.14 fgh
	*P. syringae* pv. *tomato* (*Pst2*)	1.56 j	0.81 fg	1.13 gh
*B. subtilis*	Healthy control	1.62 fgh	0.89 b–g	1.21 def
	*X. campestris* pv. *vesicatoria* (*Xcv1*)	2.05 a	1.06 a	1.43 a
	*X. euvesicatoria* (*Xe2*)	2.05 a	1.05 ab	1.42 a
	*P. syringae* pv. *tomato* (*Pst1*)	2.04 ab	1.05 ab	1.42 a
	*P. syringae* pv. *tomato* (*Pst2*)	1.99 abc	1.04 abc	1.41 a
*P. fluorescens*	Healthy control	1.68 ghi	0.88 b–g	1.19 def
	*X. campestris* pv. *vesicatoria* (*Xcv1*)	1.96 a–d	1.03 a–d	1.37 ab
	*X. euvesicatoria* (*Xe2*)	1.95 a–d	1.02 a–d	1.36 ab
	*P. syringae* pv. *tomato* (*Pst1*)	1.95 a–d	1.02 a–d	1.37 ab
	*P. syringae* pv. *tomato* (*Pst2*)	1.95 a–d	1.02 a–e	1.36 ab
*T. virdi*	Healthy control	1.69 fgh	0.88 b–g	1.20 def
	*X. campestris* pv. *vesicatoria* (*Xcv1*)	1.98 abc	1.04 abc	1.40 a
	*X. euvesicatoria* (*Xe2*)	1.97 abc	1.03 a–d	1.40 a
	*P. syringae* pv. *tomato* (*Pst1*)	1.97 abc	1.03 a–d	1.38 a
	*P. syringae* pv. *tomato* (*Pst2*)	1.97 abc	1.03 a–d	1.37 a
*T. harzianum*	Healthy control	1.68 hij	0.87 efg	1.19 e–h
	*X. campestris* pv. *vesicatoria* (*Xcv1*)	1.93 a–e	1.02 a–e	1.36 ab
	*X. euvesicatoria* (*Xe2*)	1.93 a–d	1.02 a–d	1.35 ab
	*P. syringae* pv. *tomato* (*Pst1*)	1.93a–d	1.01 a–e	1.35 ab
	*P. syringae* pv. *tomato* (*Pst2*)	1.93 efg	0.99 a–g	1.35 cde
*T. album*	Healthy control	1.67 hij	0.87 efg	1.19 fgh
	*X. campestris* pv. *vesicatoria* (*Xcv1*)	1.92b–e	0.99 a–e	1.35 abc
	*X. euvesicatoria* (*Xe2*)	1.92 b–e	0.99 a–f	1.34 abc
	*P. syringae* pv. *tomato* (*Pst1*)	1.91 cde	0.99 a–d	1.33 ab
	*P. syringae* pv. *tomato* (*Pst2*)	1.90 def	0.99 a–g	1.33 bcd
LSD	bacteria	0.05	0.03	0.04
	bioagent	0.07	0.17	0.06
	bacteria × bioagent	0.13	0.06	0.09

The letters denote Duncan’s multiple range test outcomes at *p* < 0.05 significance. Similar letters indicate non-significant treatment, while different ones denote non significance among treatments. The ‘×’ symbol refers to interaction between variables.

## Data Availability

The data presented in this study are available upon request from the corresponding author.
